# Healthy Skin

**Published:** 1855-01

**Authors:** 


**HEALTHY SKIN. A Popular treatise on the Skin and Hair. Their Preservation and Management.** By ERASMUS WILSON, F. R. S.

Mr. Wilson, in this little work now before us, has displayed an intimate knowledge of the subject of which he treats. The work is designed as a popular treatise, and the manner in which he treats the subject, places him deservedly prominent as a teacher in this department.

He treats of the nature of the scarf skin, and of the phenomena which it presents when diseased. This subject is illustrated with drawings. Those showing the animals found in the unctious matter in certain diseased conditions, as revealed by the microscope, would, from their repulsive and hideous appearance, urge those having torpid skins to renewed efforts to awaken it to activity and action, in order to avoid the generation of these "creeping things" upon the body.

In fine, every medical man should procure this little work, and examine it carefully.

Blanchard & Lea, Philadelphia, are its publishers, and if it cannot be purchased here, it can be forwarded by express at a trifling cost, or by mail.

